# Oxidative stress, histopathological and genotoxicity of copper oxide nanoparticles in *Biomphalaria alexandrina* snail

**DOI:** 10.1038/s41598-024-74439-9

**Published:** 2024-10-24

**Authors:** Mona Fathi Fol, Fathi A. Abdel-Ghaffar, Hassan Abdel-Malek Hassan, Amina Mohamed Ibrahim

**Affiliations:** 1 Zoology Department, Faculty of Science, Cairo University, Giza, Egypt; 2 Environmental Research & Medical Malacology Department, Theodor Bilharz Research Institute (TBRI), Giza, Egypt

**Keywords:** *Biomphalaria alexandrina*, CuO NPs, Histopathology, RAPD, Ecology, Ecology, Environmental sciences, Biomarkers

## Abstract

Higher usage of copper oxide nanomaterials in industrial and biomedical fields may cause an increase of these nanoparticles in aquatic environments, which could have a detrimental ecological effect. Thus, the objective of this study was to evaluate the acute toxicity of copper oxide nanoparticles on the freshwater gastropod, *Biomphalaria alexandrina*. Transmission electron microscopy, x-ray diffraction analysis and UV–VIS spectrophotometer of CuO NPs revealed a typical TEM image and a single crystal structure with average crystallite size of approximately 40 nm also, a sharp absorption band was appeared. Following exposure to sub-lethal concentrations of CuO NPs (LC_10_, 15.6 mg/l and LC_25,_ 27.2 mg/l), treated snails revealed a significant decrease (*p* < 0.05) in total antioxidant capacity, reduced glutathione contents as well as catalase, and sodium dismutase activities were significantly declined (*p* < 0.05) in comparison to the control group. Also, histopathological alterations were observed in the digestive gland, including ruptured and vacuolated digestive cells, and a marked increase in the number of secretory cells and the severity of the damage increased with rising concentrations. Furthermore, changes in RAPD profiles were detected in the treated snails. In conclusion, our research highlights the potential ecological impact of CuO NPs release in aquatic ecosystems and advocates for improved monitoring and regulation of CuO NPs industrial usage and disposal.

## Introduction

Nanomaterials are widely used in various fields of our daily life, such as biomedical, electronic, cosmetic, environmental catalysts and medical applications due to their small size and increased specific surface area^1^. Copper oxide nanoparticles have gained attention due to their multifunctional applications in industry and medicine^2^. However, according to Mandal et al.^3^, the widespread utilization of these nanoparticles makes it possible for them to be accidentally released into the aquatic environment during production, use, or disposal, resulting in detrimental effects on living organisms. *Lymnaea stagnalis* (Linneaus 1758), *Physella acuta* (Draparnaud 1822), *Potamopyrgus antipodarum* (Lamarck 1843), and *Bellamya aeruginosa* (Reeve, 1863), have been exposed to various metal-based nanomaterials, resulting in genotoxic, immunotoxic, embryotoxic, and molluscicidal effects, as well as behavioral impairments and reproductive toxicity^4^.

Invertebrates comprise approximately 95% of all animals and perform an important ecological function by passing NPs within the food industry^5^. Molluscs are found throughout along the Nile River from Aswan to Cairo^6^ and play a vital part in the ecosystem of fresh water as they provide great abundance and have good ecotoxicological characteristics for aquatic environments^7^.

The intermediate host for *Schistosoma mansoni*,* Biomphalaria alexandrina* snail has a broad distribution in Egypt’s Nile River^8^. Despite being a species of public health interest, *Biomphalaria* snails have been recognized as a potential biomonitoring of environmental quality due to its sensitivity to pollutants, such as metals and nanoparticles, as well as its ease of maintenance and reproduction in laboratories due to its small size, high egg production, and rapid development^9^. Environmental biomonitoring could help to elucidate the harmful effects of heavy metal on the biological systems of the surrounding organisms^10^. Previous studies have shown that high metal NPs accumulation in freshwater gastropods could induce toxic effects^11^, such as oxidative stress, which may result in changes in the development and reproduction of gastropods^12^. Kandeil et al.^13^ reported that both ZnO forms (bulk and nano) markedly changed the reproductive performance of the freshwater snail, *Helisoma duryi.* Jeyavani and Vaseeharan^14^ showed the toxic effects of environmental predominant microplastics and ZnO nanoparticles in freshwater snail *Pomaceae paludosa* Wang and Liu^15^ found that chronic exposure to AgNPs induces bioaccumulation, immune system impairments, reduction in reproductive output and condition index in the freshwater gastropod *Lymnaea stagnalis.* Also, Rodrigues et al.^16^ stated that iron oxide nanoparticles and ferric chloride induce inflammatory response and histopathological changes in gonad of the freshwater snail *Biomphalaria glabrata.*

Therefore, the current research aimed to evaluate the acute toxicity of CuO NPs on *B. alexandrina* snails using oxidative, histopathological, and molecular parameters.

## Results

### Characterization of CuO NPs

**Fig. 1:**
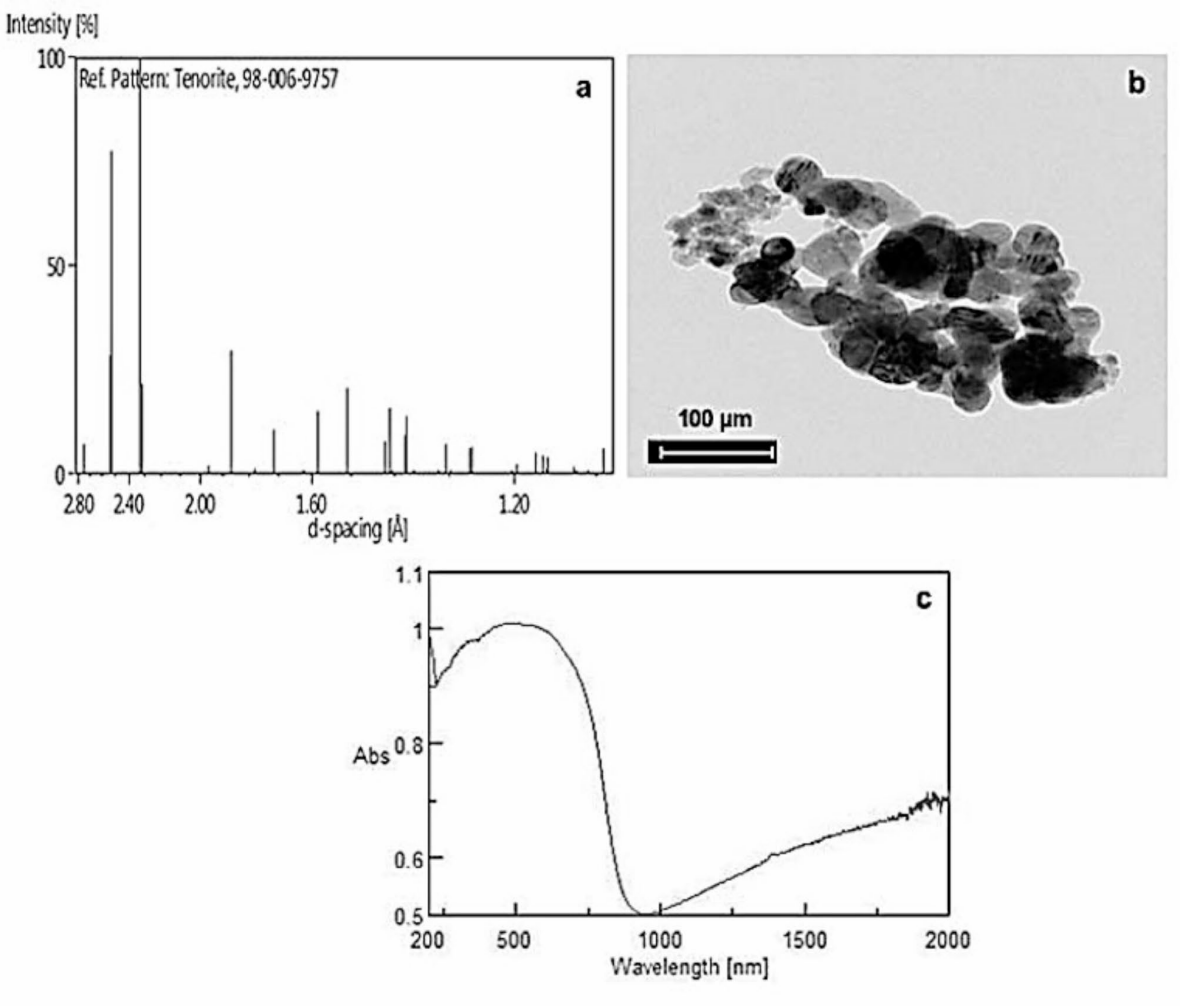
Characterization of CuO NPs, (**a**) XRD pattern (**b**) TEM image (**c**) UV–visible spectrum.

X-ray diffraction pattern confirmed the single crystal structure of CuO NPs at 196 K and room temperature (Fig. 1a). No impurity-specific peaks were detected, indicating that high-quality CuO NPs were synthesized. In addition, a typical transmission electron microscopy (TEM) image of CuO NPs revealed that most of the particles have polygonal shapes with smooth surfaces and that their average diameter is 40 ± 5 nm (Fig. 1b). The UV–VIS spectrophotometer showed a sharp absorption band (Fig. 1c).

### Release of Cu^2+^ concentration

Copper ion concentrations were determined in test water to be 0.0002, 0.0051, and 0.016 mg/l for the control, and the two sub-lethal concentrations of CuO NPs solution, respectively. The recorded results revealed that Cu^2+^ ion increased along with rise in CuO NPs concentration.

### Effect of CuO NPs on the oxidative stress of * B. alexandrina*

**Fig. 2:**
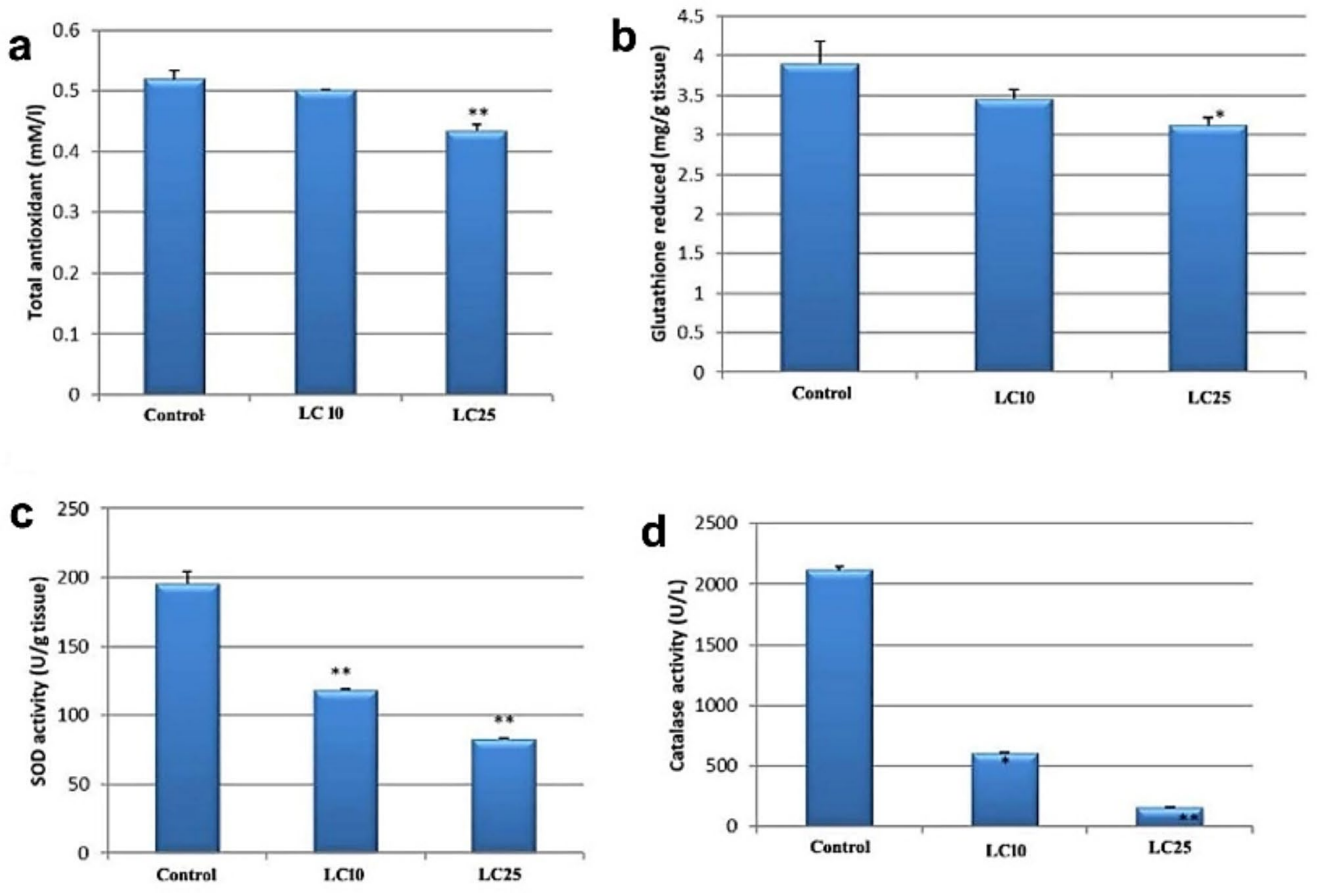
Effect of CuO NPs on *B. alexandrina* (**a**) TAC (**b**) GSH (**c**) SOD (**d**) CAT.

Snails exposed to LC_10_ and LC_25_ of CuO NPs exhibited a significant decrease (*p* < 0.05) in TAC and GSH contents as well as a significant decline in SOD and CAT activities compared to the control group (Fig. 2).

### Histopathological observations

**Fig. 3:**
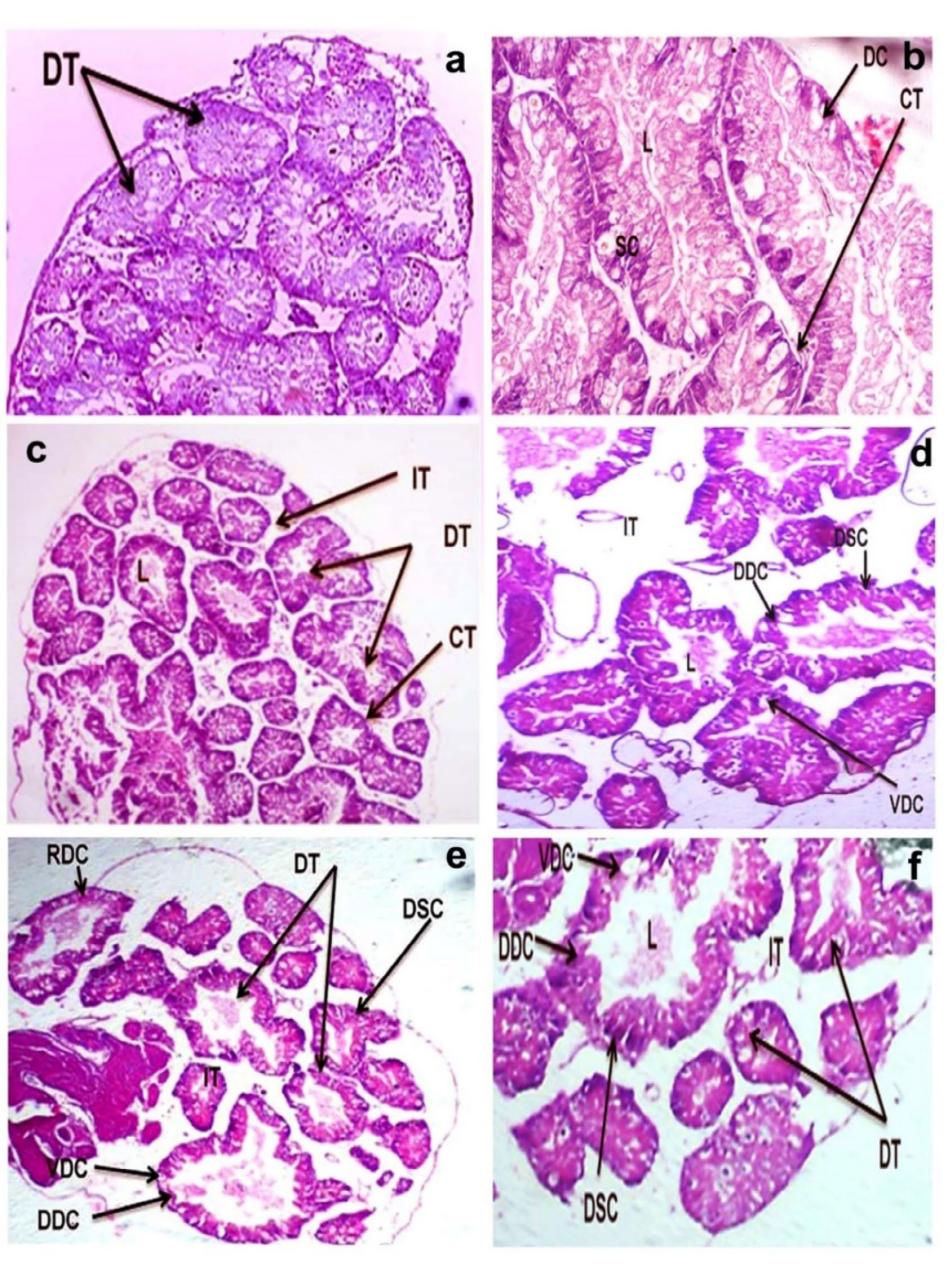
Photomicrograph of the digestive gland of *B. alexandrina* snail showing (**a**) Normal digestive gland of control snails with digestive tubules (DT), (H&E X 40). (**b**) A higher magnification of normal digestive gland of control *B. alexandrina* showing digestive tubule with digestive cells (DC) and secretory cells (SC) surrounded by loose connective tissue (CT) and lumen (L), (H&E X 400). (**C**) Snails exposed to LC_10_ (15.6 mg/l) of CuO NPs displaying. Degeneration and shrinkage of some tubules (DT), increase in inter tubular space (IT) and connective tissues (CT) between tubules (H&E X 40). (**d**) A higher magnification of digestive gland showing vacuolated digestive cell (VDC), degeneration of digestive cell (DDC) and secretory cells (DSC), and an increase in the inter tubular space (IT) and lumen (L), (H&E X 100). (**e**) Snails exposed to LC_25_ (21.18 mg/l) of CuO NPs showing rupture of some tubules (DT), degeneration of digestive cell (DDC) and secretory cells (DSC), vacuolated digestive cell (VDC), and increase in the inter tubular space (IT) and lumen (L), (H&E X 40). (**f**) Higher magnification of the digestive gland showing congestion of the central lumen, and pyknosis of nuclei (arrow) with high stained cytoplasmic granules * IT* intertubular space, * DDC* degenerated digestive Cell, * DSC* Degenerated secretory cells, * VDC* vacuolated digestive cell, * L* lumen, (H&E X 100).

The digestive gland of control snails consists of tubular glands lined with a single layer of digestive cells and secretory cells (Fig. 3a, b). At LC_10_, most of these cells became vacuolated, degenerated, and ruptured, and the lumen expanded (Fig. 3c, d). Most cells at LC_25_ of CuO NPs lost their identical shape due to the dissolution of their membrane, and the tips of some digestive cells were ruptured (Fig. 3e, f).

**Fig. 4:**
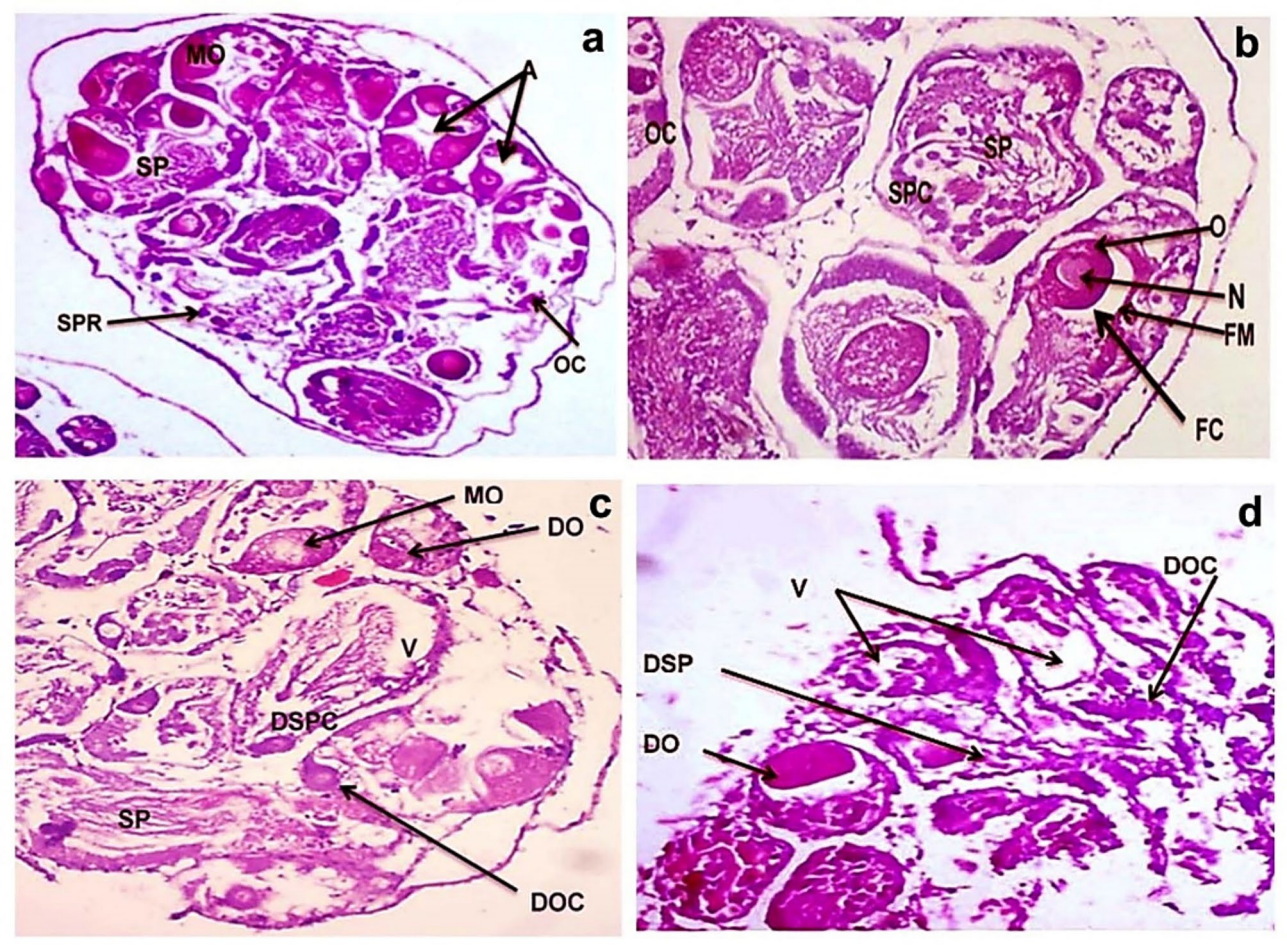
Photomicrograph of hermaphrodite gland of *B. alexandrina* snail showing (**a**,**b**) control hermaphrodite gland displaying female gonads with normal acini (A), and oocytes (OC), mature ovum (MO). (**b**) The ovum (O) surrounded by follicular membrane (FM) and follicular cavity (FC) and a large nucleus (N) appeared, male seminiferous tubule with a wide lumen contains spermatocytes (SPC) and spermatids (SP) (H&E X 100). (**c**) hermaphrodite gland after exposure to LC_10_ of CuO NPs revealing degenerations in oocytes, ova and spermatocytes. Most of the reproductive cells are empty and vacuolated (V) (H&E X 40). * MO* mature ova, * DOC* degenerated Oocyte, * SP* spermatids, * V* Vacuole, * DO* degenerated ovum, * DSPC* degenerated spermatocytes, (**d**) hermaphrodite gland after exposure to LC_25_ of CuO NPs exhibiting severe reduction in size where the acini were shrunken, and the tubules compacted together. Some tubules were degenerated and others being highly affected, Ova and oocytes are damaged, lumen of the tubules is concentrated with the degenerated spermatozoa. * DO* degenerated ovum, * DOC* degenerated oocyte, (H&E X 100).

Concerning the hermaphrodite gland of control snails, the male reproductive cells were differentiated into clusters forming primary and secondary spermatocytes, and the female oogenic cells filled the acinar lumen and the mature ova were surrounded by follicular membrane (Fig. 4a, b). Exposing snails to LC_10_ of CuO NPs revealed shrinkage and destruction of the sperms and degeneration of the eggs (Fig. 4c), the great damage in gonadal cells occurred at LC_25_, where eggs lost their shapes and degenerated. The number of sperms decreased, and connective tissue was replaced by vacuoles (Fig. 4d).

### Genotoxicity of CuO NPs on* B. alexandrina*

**Fig. 5:**
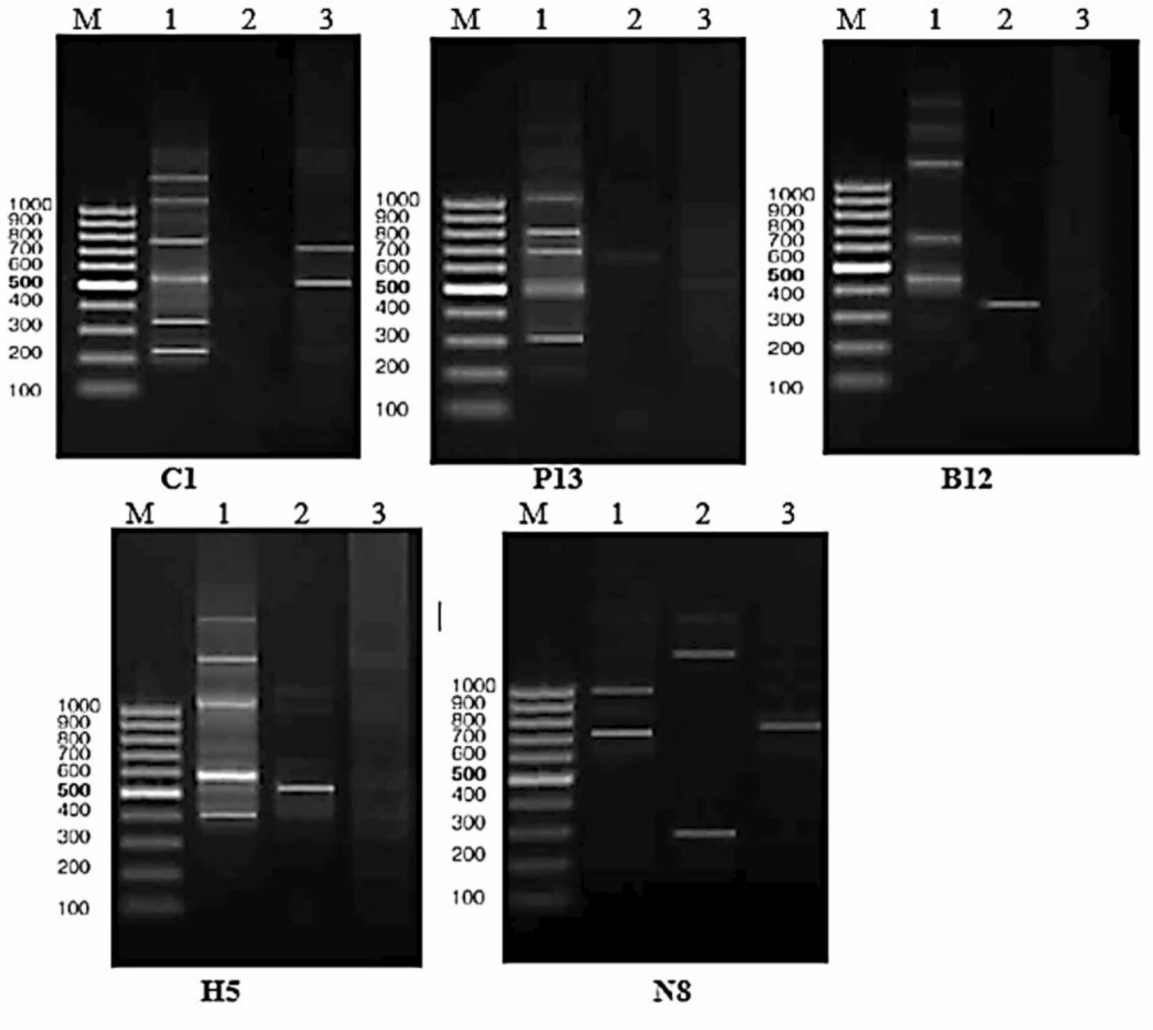
RAPD profile of *B. alexandrina*, Lanes; M: 100 bp plus DNA ladder, 1: control group, 2: LC_10_ (15.6 mg/l), 3: LC _25_ (27.2 mg/l) of CuO NPs.

Six primers were used to perform individual agarose gel amplifications of extracted DNA from the head-foot of *B. alexandrina* to determine the genotoxicity of CuO NPs. According to the RAPD-PCR profiles, only five of the six primers produced 26 distinct and reproducible bands ranging in size from 100 to 2000 bp. The highest number of PCR fragments were obtained with primers C1 and H5 (six bands), while fewer fragments were obtained with primer B12 (four bands) (Fig. 5). The difference between control and treated snails is reflected by the appearance of new bands and the disappearance of the normal bands, as shown in (Table 1). In addition, the percentage of genome stability was calculated based on the analysis of RAPD profiles, which revealed 19 and 5, at LC_10_ and LC_25_ respectively, and decreased as CuO NPs concentration increased (Table 2). Genotoxicity was detected between control and treated snails based on a difference in RAPD profile and a reduction in GTS.

**Table 1:** RAPD primers used to evaluate genotoxicity of CuO NPs against *B. Alexandrina*.

Primer	Primer sequence (5´→ 3´)	Length (bp)	Total bands	Polymorphic bands	% of polymorphism
C1	TTCGAGCCAG	10	6	4	66.67
P13	GGAGTGCCTC	10	5	5	100
B12	ACCTCAGCTC	10	4	4	100
H5	CCTTGACGCA	10	6	6	100
N8	AGTCGTCCCC	10	5	5	100
Total bands			26	24	92.3

**Table 2:** Summarizes the changes detected in RAPD profile of control and treated *B. alexandrina* snail following exposure to sub-lethal concentrations (LC_10_, 15.6 mg/l and LC_25,_ 27.2 mg/l) of CuO NPs.

Primer number	Primer	Control	Treated			
			LC_10_		LC_25_	
			*p*	D	*p*	d
1	C1	6	0	6	2	4
2	P13	5	0	5	0	5
3	B12	3	1	3	0	3
4	H5	5	1	5	0	5
5	N8	2	2	2	1	2
	Total bands	21	4	21	3	19
	A		25		22	
	a\n		1.19		1.05	
	1-(a/n)		0.19		0.05	
	% GTS		19		5	
**p** is appearance band in treated sample, **d** is disappearance band in treated sample **a** is polymorphic profile (a = d + p)						

## Discussion

The usage of nanoparticles in commercial and biomedical applications has increased recently^17,18^. Because of their remarkable physicochemical features, metal oxide NPs are extremely appropriate and desired for numerous consumer products and industrial technologies^19^. Metal oxide nanoparticles, such as CuO NPs, have received particular attention due to their antibacterial, anticancer, and antioxidant activity^20^. Since the aquatic ecosystem receives wastewater from domestic and industrial sources, it is being targeted with nanoscale environmental remediation techniques^21^. The present investigations revealed that copper oxide nanoparticles were toxic to *B. alexandrina* snails after 24 h of exposure at both sub-lethal concentrations. These findings are consistent with those of Ibrahim et al.^22^, who discovered a reduction in the growth and reproductive rates of *B. alexandrina* exposed to CuO NPs, as well as a drop in egg viability. Also, Gansen et al.^23^ found that CuO NPs were harmful to the freshwater crustacean *Daphnia magna* with LC_50_ values ranging from 0.06 to 9.80 mg/l. Similarly, Abd El-Atti et al.^24^ verified the toxicity of CuO NPs on the crayfish *Procambarus clarkii*, discovering the mortality rates were 0%, 6.7%, and 36.7% following exposure to 25, 125, and 250 mg/l of CuO NPs, respectively. Furthermore, Zhao et al.^25^ claimed that CuO NPs were more harmful than CuO BPs in juvenile crap (*Cyprinus carpio*).

Nanoparticles caused excess production of reactive oxygen species (ROS) triggering a variety of biological reactions that damaged proteins and DNA^23^. According to Bhagat et al.^26^, oxidative biomarkers are valuable indicators to investigate nanotoxicity. Many studies demonstrated that CuO NPs could induce ROS and cause oxidative damage^23,24^. Also, Chung et al.^27^ concluded that CuO NPs produced oxidative stress that led to apoptosis in the cells.

Total antioxidant capacity describes the ability of molecules to scavenge free radicals^28^. Catalase and SOD are antioxidant enzymes that neutralize detrimental toxic effects and are considered the main defense response against cellular oxidative damage^29^. Regarding TAC and GSH contents, our results showed a significant decrease in tissues of *B. alexandrina* snails following exposure to CuO NPs. Also, CAT and SOD activities revealed a significant decrease in snail’s tissues at both concentrations compared to control group. This is in accordance with Ganesan et al.^23^ who exhibited that CuO NPs might cause oxidative stress in aquatic organisms by inhibiting CAT, SOD and GSH activities. Similarly, Abdel-Khalek et al.^30^ showed a reduction in GSH content in tissues of the Nile Tilapia; *Oreochromis niloticus* after exposure to LC_50_ of CuO NPs. On the same line, Abd El-Atti et al.^31^ revealed that exposing adult crayfish to 25, 125, and 250 mg/l CuO NPs for 28 days caused a significant drop in levels of GSH, total lipids and total proteins resulting in several biochemical alterations in *Procambraus clarkii.* Likewise, Abdel-Khalek et al.^30^ reported that CAT and SOD activities were significantly inhibited in Nile Tilapia; *Oreochromis niloticus* liver tissue exposed to 1/10 and 1/20 LC_50_ 96 h of CuO NPs. Correspondingly, Atli et al.^32^ found a decrease in CAT activity in tissue of copper heavy metal-treated fishes.

The histopathology technique is a reliable and useful tool for determining the toxicity of environmental contaminants on aquatic animal organs^33^. Because of its function in pollutant material detoxification, the digestive gland of molluscs is one of the target organs in toxicological investigations^34^. Copper oxide nanoparticles caused histological changes in the digestive gland throughout the current study, and the severity of the damage increased with rising concentrations. The most prominent severe damage in the digestive cells was the presence of a great loss of its identical shape. After exposure to LC_10_ of CuO NPs, tips ruptured and most of these cells were degenerated, secretory cells multiplied, and the connective tissue between digestive tubules diminished. The size of the digestive tubules and the vascular connective tissue were extremely reduced, and the lumen was expanded, when the snails were exposed to LC_25_ of CuO NPs. The changes in the digestive cells were more severe with marked apoptosis. The epithelial cells in most tubular regions deteriorated and lost their cell borders, with the secretory cells being particularly impacted. These observations agree with Sawasdee et al.^35^ who displayed destruction of tubules, vacuoles, and necrosis of digestive gland of *Marisa cornuarietis* snail after exposure to copper and lithium. Correspondingly, Abd El-Atti et al.^31^ found that the hepatopancreas of the adult crayfishes exhibited lumen dilatation and vacuolation following exposure to 25, 125 and 250 mg/l of CuO NPs. They concluded that CuO NPs induce many histopathological alterations in *Procambrus clarkii* due to the bioaccumulation of copper in gills, hepatopancreas and muscles. Similarly, Hamdi et al.^36^ showed severe damage in the digestive gland of *B. alexandrina* after exposure to zinc metal. The digestive cells lost their identical shapes with numerous vacuoles, and had damaged tips, while the secretory cells developed a denser color and some of them ruptured. In addition, histopathological examinations of the hermaphroditic gland of *B. alexandrina* snails revealed marked degenerative changes, losses of connective tissues, deformation of sperms, and disintegration of eggs following CuO NPs exposure. At LC_25_, severe damage, evacuation, and destruction of gonadal cells were induced. The connective tissue between the acini was dissolved and replaced by vacuoles. Ova and sperms lost their shapes and degenerated. Moreover, most of the spermatogenic stages disappeared. These results agree with Ibrahim et al.^37^ who reported great damages in the gonadal cells of *B. alexandrina* with degeneration of mature ova, spermatocytes, oocytes, and sperms following exposure to Ce_2_O_3_/MNCs. Similarly, Abdel-Khalek et al.^38^ demonstrated that CuO NPs were more effective at penetrating fish tissues, as confirmed by histopathological examinations that revealed alterations ranging from adaptation responses to persistent tissue damage. Also, Saad et al.^39^ found that snails exposed to LC_25_ of CuO NPs suffered from great damage in gonadal cells as evidenced by the degeneration of mature ova and sperms and the evacuation of many gonads’ cells. Moreover, Rawi et al.^40^ established that ZnSO_4_ caused destructive alterations and vacuoles in the gonadal cells of *B. alexandrina*.

Reactive oxygen species (ROS) can induce DNA strand breaks and affect gene expression^41^. However, little is known regarding the genotoxicity of nanoparticles, particularly in aquatic molluscs. RAPD analysis is non-radioactive, non-intrusive, and capable of detecting numerous varieties of DNA damage and mutations in any organism^42,43^. This technique has been successfully used to investigate various contaminants affecting population genetics^44,45^. In comparison to control snails, the results of RAPD profile of treated snails revealed the disappearance of normal bands and the appearance of new one with percentage of polymorphism 92.3%, indicating that CuO NPs cause DNA damage. On the same line, Atienzar et al.^46^ reported that the loss and presence of bands reduce the stability of genomic DNA led to damage of DNA. Similarly, Abd El-Atti et al.^31^ concluded that high concentrations of CuO NPs (125 and 250 mg/l) caused nuclear DNA damage in the exposed crayfishes *P. clarkii*. Likewise, Nikdehghan et al.^47^ reported that CuO NPs exposure induced micronuclei formation and DNA damage in *Cyprinus carpio*. Similarly, Mosa et al.^48^ indicated that Cu NPs caused genomic alterations in *Cucumis sativus* using RAPD-PCR technique. Also, Shaldoum et al.^49^ confirmed the genotoxic and mutagenic potential of Cu_2_O NPs and CuSO_4_ on *B. alexandrina* snails. Additionally, He et al.^50^ found that copper oxide nanoparticles induce oxidative DNA damage and cell death. Consequently, Ibrahim and Sayed^51^ linked between the genotoxic effects of molluscicides with the results of the antioxidant parameters, where the increase of free radicals in organisms after exposure to chemical contaminants or stressors could deteriorate antioxidant defensive system by ROS and damaged DNA.

The genomic templates stability (GTS, %) is a percentage value that reflects variations in the PCR amplification profile of a test sample compared to a control one. In the present investigation, the percentage of genome stability was calculated from the analysis of RAPD profiles revealed 19 and 5 at LC_10_ and LC_25_ respectively, which decreased as concentration of CuO NPs increased. In accordance Kumar et al.^52^ reported that GTS in treated *Cyprinus carpio* decreased as potassium dichromate concentrations and exposure duration increased.

## Conclusion

The current study revealed the toxicity of CuO NPs on the freshwater snail, *B. alexandrina* via declines in antioxidant enzymes and alterations in the hermaphrodite and digestive glands, as well as changes in the RAPD profile causing genotoxicity. Thus, ubiquitous production and use of CuO NPs may result in unintended environmental releases, especially in aquatic ecosystems. To control their use and discharge, additional research is required to determine the environmental impact of CuO NPs in the Nile River.

## Materials and methods

### Snails’ collection

*Biomphalaria alexandrina* snails (8–10 mm) were obtained from Medical. Malacology Laboratory, Theodor Bilharz Research Institute (TBRI), Giza, Egypt, and kept in plastic aquaria (16 × 23 × 9 cm), filled with dechlorinated tap water (30 snails/L) and covered with glass plates. They were maintained in air-conditioned rooms at 25 °C and fluorescent light was reflected 30 cm over them during day. The snails were fed blue green algae (*Nostoc muscorum*) and oven dried lettuce leaves.

### Characterization of CuO NPs

Copper oxide nanoparticles powder with average particle size < 50 nm was purchased from NanoTech Egypt for Photo-Electronics, Egypt. The structure of CuO NPs was examined using a high-resolution transmission electron microscope (FETEM, JEM-2100 F, JEOL Inc., Japan) produced by Nanotechnology and Advanced Material Central Lab (NAMCL), Agriculture Research Center (ARC). Two imaging modes were used: bright field at 200 kV electron accelerating voltage utilizing lanthanum hexaboride (LaB6) electron source gun and diffraction pattern imaging. The X-ray diffraction pattern was used to determine the crystalline nature of CuO NPs. Also, the optical absorption of CuO NPs suspension was measured using a double beam UV–Vis-NIR spectrophotometer (Varian-Cary 5000) in the wavelength range of 200–800 nm at room temperature.

### Copper ion analysis in tested water

The copper ion (Cu2^+^) was tested in the solution of CuO NPs by a flame atomic absorption spectrophotometer (ICP-MS; ELAN DRC II, PerkinElmer, Waltham, MA, USA) as described by Richardson^53^.

### Determination of acute toxicity of CuO NPs

A stock solution of 1000 mg/l CuO NPs was prepared by dispersing CuO NPs in distilled water and incubating for 90 min in an ultrasonic bath; a further 20 min of sonication was performed shortly before usage. Series concentrations of copper oxide nanoparticles (10, 15, 20, 25, 50, 75, 100, and 200 mg/l) were prepared in aquaria of 100 ml of dechlorinated water. For each concentration, 10 snails were put in a beaker. Another ten snails were dipped in dechlorinated water as control. Three replicates were used for each concentration for 24 h. Snails were removed, washed, and then transferred to dechlorinated water for another 24 h of recovery according to WHO^54^. The lethality of CuO NPs on *B. alexandrina* snails was detected by Probit analysis using mortality data for each concentration after 24 h from the beginning of the experiment. Then, two sub-lethal concentrations of CuO NPs were selected.

### Experimental design

Thirty snails were exposed to each sub-lethal concentration of CuO NPs, LC_10_ (1.56 mg/l) and LC_25_ (2.72 mg/l), and a corresponding control group were kept in dechlorinated water for 24 h exposure, then the snails were removed from the experimental test solutions and transferred to clean dechlorinated water for another 24 h of recovery/week. For each concentration, three copies were made and the experiment was repeated for two weeks.

### Oxidative stress markers

Soft tissues of control and CuO NPs treated groups were weighed and homogenized for 5 min in phosphate buffer at a weight-to-volume ratio of 1:10 using a glass homogenizer. Then homogenates were centrifuged at 4 °C for 15 min at 3000 rpm, and the supernatants were stored at -20 °C until use. Supernatant was used for the determination of the total antioxidant capacity (TAC) according to Koracevic et al.^55^. Glutathione reduced (GSH), the primary intracellular thiol used by cells as a marker for antioxidant protection, was estimated as described by Beutler et al.^56^. Catalase activity (CAT) is utilized for protection against H_2_O_2_-induced stress and was detected following the method of Aebi^57^. Also, the activity of superoxide dismutase (SOD) was determined using the method described by Nishikimi et al.^58^ All oxidative biomarkers were assessed using Bio Diagnostic kits, Egypt.

### Histopathological examination

The hermaphrodite and digestive glands of control and CuO NPs treated snails were gently separated and washed with saline then fixed in 10% buffered formalin. Samples were dehydrated in ascending grades of alcohol, cleared with xylene, and embedded in paraffin. Five µm sections were cut and stained with hematoxylin and eosin (H& E). Sections were examined and photographed using a LEICA DM 750 microscope equipped with a LEICA ICC 50 HD camera.

### DNA extraction and RAPD-PCR analysis

Soft parts removed after dissection of snails, then preserved in 100% ethyl alcohol at -20 °C until used. Random amplified polymorphic DNA (RAPD) technique was performed on DNA extracted from the head-foot of both control and treated snails using Qiagen Dneasy tissue kit (Valencia, CA, USA) according to the manufacturer’s manual. Six random primers were used to amplify DNA samples (C1, P13, B12, H5, N8 and P8).

Amplifications were performed by modifying the protocol reported by Williams et al.^59^. The 50 ul PCR mixture contained 25 ng of template DNA, 1.5 unit of Taq polymerase, 10 µ MdNTPs, 10 pM primers and 2.5 ul of 10x PCR buffer: MyTaq HS Red Mix, 2x (Bioline). Each amplification reaction was performed using a single primer and repeated twice to verify band auto similarity (Perez et al. 1998). Amplifications were performed in T-personal thermal cycler (Techne, TC-3000G), programmed for 45 cycles of 94 °C for 1 min, 35 °C for 1 min, and 72 °C for 1 min. An initial denaturation step (3 min, 94 °C) and a final extension holding (10 min, 72 °C) were included in the first and last cycles, respectively. Ten ul of the reaction products were resolved by 2% agarose gel electrophoresis at 85 volts in 1x TAE (Tris-acetate-EDTA) buffer. Ethidium bromide was used to stain the gel which was photographed with Gel Documentation System. Population-specific fragments were detected using Gene Ruler 100 kb Plus DNA Ladder (Fermentas, Canada) to compare the amplified products.

### Estimation of genomic template stability

The polymorphic pattern generated by RAPD-PCR profiles using the selected primers allowed the calculation of genomic template stability percent (GTS %) as follows: GTS= (1-a/n) x 100.

Where a, is the average number of polymorphic bands and n the number of total bands in the control group. Polymorphisms in RAPD profiles included disappearance of bands and appearance of a new one with respect to the control profile. To compare the sensitivity of genomic template stability, changes in these values were calculated as a percentage of their control.

### Statistical analysis

Data were analyzed by one-way ANOVA using Statistical Processor Systems Support, SPSS software, version 20, followed by Least Significant Difference post hoc tests to compare group means. Values are expressed as mean ± SE, and *p* < 0.05 was considered significant.

## Electronic supplementary material

Below is the link to the electronic supplementary material.

Supplementary Material 1.

Supplementary Material 2.

Supplementary Material 3.

Supplementary Material 4.

Supplementary Material 5.

## Data Availability

The data analyzed are saved during the current study and available from the corresponding author on request.
