# Supplementary figures and images for: Oxidative stress, histopathological and genotoxicity of copper oxide nanoparticles in Biomphalaria alexandrina snail
Source: Sci Rep. 2024 Oct 24;14:25187. doi: 10.1038/s41598-024-74439-9 (PMC11502862; doi:10.1038/s41598-024-74439-9)

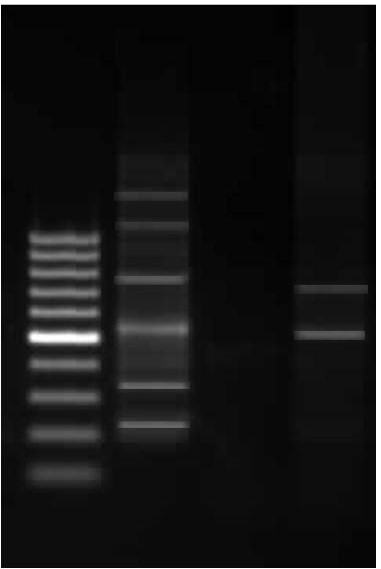

Supplement: Supplementary file 1 — Supplementary Material 1. [file 41598_2024_74439_MOESM1_ESM.jpg]

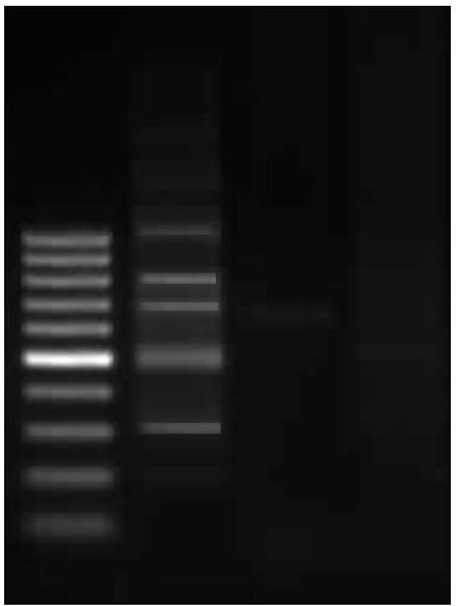

Supplement: Supplementary file 2 — Supplementary Material 2. [file 41598_2024_74439_MOESM2_ESM.jpg]

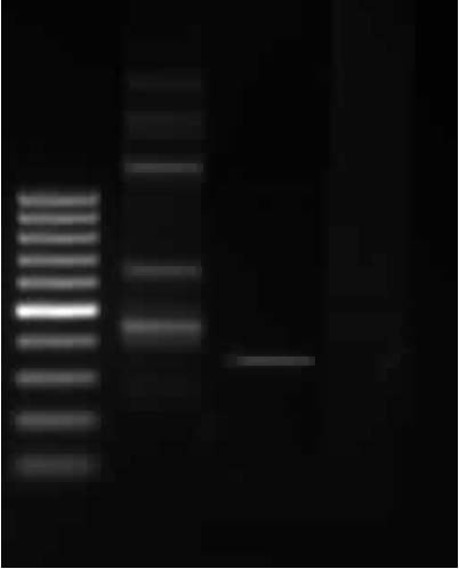

Supplement: Supplementary file 3 — Supplementary Material 3. [file 41598_2024_74439_MOESM3_ESM.jpg]

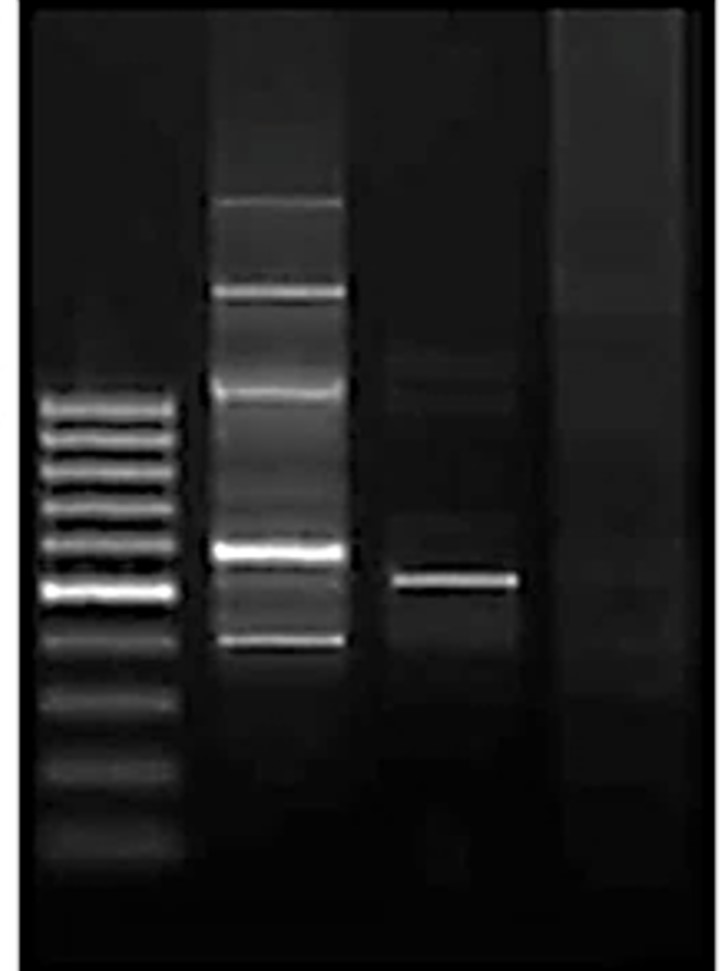

Supplement: Supplementary file 4 — Supplementary Material 4. [file 41598_2024_74439_MOESM4_ESM.jpg]

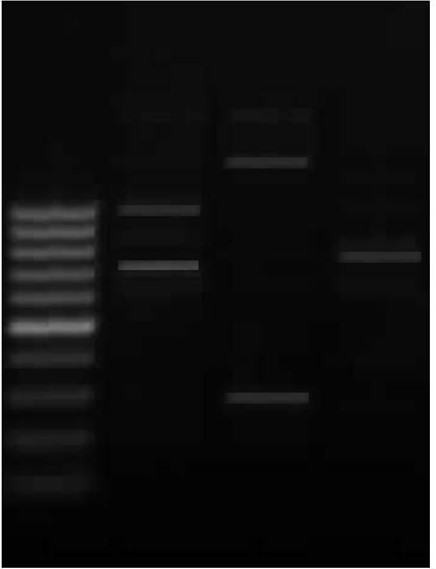

Supplement: Supplementary file 5 — Supplementary Material 5. [file 41598_2024_74439_MOESM5_ESM.jpg]
